# Aggression Following Traumatic brain injury: Effectiveness of Risperidone (AFTER): study protocol for a feasibility randomised controlled trial

**DOI:** 10.1186/s13063-018-2601-z

**Published:** 2018-06-21

**Authors:** Shoumitro Deb, Verity Leeson, Lina Aimola, Mayur Bodani, Lucia Li, Tim Weaver, David Sharp, Mike Crawford

**Affiliations:** 1 Imperial College London, Department of Medicine, Division of Brain Sciences, Centre for Psychiatry, 7th Floor Commonwealth Building, Hammersmith Hospital Campus, Du Cane Road, London, W12 0NN UK; 2 Department of Neuropsychiatry, Kent and Medway NHS and Social Care Partnership NHS Trust, Daren House, Sevenoaks, Kent, TN13 3PG UK; 3 Imperial College London, Department of Medicine, Division of Brain Sciences, The Computational, Cognitive and Clinical Neuroimaging (C3NL), Hammersmith Hospital Campus, Du Cane Road, London, W12 0NN UK; 4 Department of Mental Health Research, School of Health and Education, Middlesex University, London, UK

**Keywords:** Traumatic brain injury, Aggression, Pharmacological intervention, Risperidone, Feasibility RCT

## Abstract

**Background:**

Traumatic brain injury (TBI) is a major public health concern and many people develop long-lasting physical and neuropsychiatric consequences following a TBI. Despite the emphasis on physical rehabilitation, it is the emotional and behavioural consequences that have greater impact on people with TBI and their families. One such problem behaviour is aggression which can be directed towards others, towards property or towards the self. Aggression is reported to be common after TBI (37–71%) and causes major stress for patients and their families. Both drug and non-drug interventions are used to manage this challenging behaviour, but the evidence-base for these interventions is poor and no drugs are currently licensed for the treatment of aggression following TBI. The most commonly used drugs for this purpose are antipsychotics, particularly second-generation drugs such as risperidone. Despite this widespread use, randomised controlled trials (RCTs) of antipsychotic drugs, including risperidone, have not been conducted. We have, therefore, set out to test the feasibility of conducting an RCT of this drug for people who have aggressive behaviour following TBI.

**Methods/design:**

We will examine the feasibility of conducting a placebo-controlled, double-blind RCT of risperidone for the management of aggression in adults with TBI and also assess participants’ views about their experience of taking part in the study.

We will randomise 50 TBI patients from secondary care services in four centres in London and Kent to up to 4 mg of risperidone orally or an inert placebo and follow them up 12 weeks later. Participants will be randomised to active or control treatment in a 1:1 ratio via an external and remote web-based randomisation service. Participants will be assessed at baseline and 12-week follow-up using a battery of assessment scales to measure changes in aggressive behaviour (MOAS, IRQ) as well as global functioning (GOS-E, CGI), quality of life (EQ-5D-5L, SF-12) and mental health (HADS). We will also assess the adverse effect profile with a standard scale (UKU) and collect available data from medical records on blood tests (serum glucose/HbA1c, lipid profile, prolactin), and check body weight and blood pressure. In addition completion of the MOAS and a check for any new or worsening side-effect will be completed weekly and used by the prescribing clinician to determine continuing dosage. Family carers’ wellbeing will be assessed with CWSQ. Service use will be recorded using CSRI. A process evaluation will be carried out at the end of the trial using both qualitative and quantitative methodology.

**Discussion:**

Aggressive behaviour causes immense distress among some people with TBI and their families. By examining the feasibility of a double-blind, placebo-controlled RCT, we aim to discover whether this approach can successfully be used to test the effects of risperidone for the treatment of aggressive behaviour among people with aggression following TBI and improve the evidence base for the treatment of these symptoms. Our criteria for demonstrating success of the feasibility study are: (1) recruitment of at least 80% of the study sample, (2) uptake of intervention by at least 80% of participants in the active arm of the trial and (3) completion of follow-up interviews at 12 weeks by at least 75% of the study participants.

**Trial registration:**

ISRCTN30191436. Registered on 19 December 2016.

**Electronic supplementary material:**

The online version of this article (10.1186/s13063-018-2601-z) contains supplementary material, which is available to authorized users.

## Background

Traumatic brain injury (TBI) is a major public health concern in the UK. Each year, 1.4 million people attend hospitals in England and Wales with a recent TBI (about 80% have mild TBI) of them around 200,000 people are admitted to hospital [1, 2]. More and more patients with TBI are surviving longer now because of improved medical and surgical treatment of their injuries. However, it has been shown that long-term consequences are common after TBI of all severity. Thornhill and colleagues (2000) [3] showed that, of patients admitted to hospital with TBI, 86% of those who had sustained a severe TBI, 62% of those who had sustained a moderate TBI and 55% of those who had sustained a mild TBI had physical and emotional problems 1 year after their injury. In the first-ever comprehensive assessment of neuropsychiatric consequences after TBI we found that 32% of patients aged 18–65 years had suffered from a psychiatric disorder in the year following their injury, compared to 16% of the general population [4–7].

Of all the neurobehavioural problems following TBI, aggression remains the most challenging. The long-term prevalence of aggressive behaviour following mild to severe TBI varies between 37% and 71% according to different studies [8, 9]. Aggression affects quality of life of both the patients and their family carers and remains the most difficult problem clinically to manage. Both pharmacological and non-pharmacological interventions are used to manage this difficult behaviour in practice. However, scientific evidence to suggest that any intervention works is lacking. Our systematic review [10] and a recent Cochrane review [11] showed lack of good-quality randomised control trials (RCTs) in this field. While very small-scale RCTs (involving only 21, 11 and 4 participants, respectively) of high doses of propranalol have been conducted, concerns about adverse events of these drugs at a high dose means that they are not widely used in clinical practice. The most commonly used groups of drugs in TBI are antipsychotics and mood stabilisers/antiepileptic medications [12]. Among the antipsychotics, the new generation of drugs, such as risperidone, quetiapine, olanzapine and aripiprazole, are used more often than the old generation of antipsychotics [13]. However, because of lack of evidence and in the absence of any national/international guidelines, clinicians tend to choose antipsychotics based on their, often limited, personal experience and anecdotal evidence.

As risperidone is widely used in people with TBI for the management of aggression and, is the only drug for which there is some evidence of effectiveness in the management of aggression in people with dementia, autism spectrum disorder (ASD) and intellectual disabilities (ID), but as no such RCTs exist involving people with TBI [14–17], we have chosen low-dose risperidone as the active intervention drug for our placebo-controlled, feasibility, double-blind RCT for people with TBI. Data from this feasibility study will allow us to develop a full-scale RCT in the future.

In keeping with MRC guidelines on the evaluation of complex interventions, we will integrate a process evaluation within this feasibility study [18]. We will explore participants’ beliefs about the impact of the intervention (including both positive and negative effects), mechanisms of action, and factors that facilitate or hinder its successful delivery.

## Aims and objectives

(1) to improve the management of aggression among people with TBI by providing evidence for a particular pharmacological intervention, (2) to assess outcome at 12 weeks using the Modified Overt Aggression Scale (MOAS) [19] as the primary outcome measure to assess aggression in order to estimate sample size for future full RCT, and gauge the potential recruitment and dropout rates, (3) to conduct a multi-centre, parallel-design, placebo-controlled (1:1 ratio), double-blind RCT of risperidone for the management of aggression in adults with TBI in order to assess feasibility for a substantive, full-scale, definitive RCT in future and (4) to carry out a process evaluation at the end of the RCT follow-up.

## Methods/design

The study will randomise up to 50 adults with TBI and aggressive behaviour into risperidone and placebo groups in equal numbers. The study is a four-centre, parallel-design, double-blind, flexible-dose RCT. The current study will allow us to assess a number of areas of feasibility, which will determine the viability of undertaking a full-scale RCT and ensure appropriate methodological design.

**Inclusion criteria**

(1) aged between 18 and 65 years, (2) a confirmed clinical diagnosis of TBI which occurred at least 6 months prior to recruitment, evidenced as a rating of moderate/severe or mild (probable) based on Mayo Clinic criteria [20], (3) referred to the clinician for the management of aggression and for whom the clinician is considering a pharmacological intervention for this problem after investigating and addressing physical, psychological and social triggers and (4) competent and willing to provide written, informed consent.

**Exclusion criteria**

(1) suffering from post-traumatic amnesia (PTA), (2) co-morbid serious mental illness, such as schizophrenia and other psychoses, bipolar disorder, major depressive disorder, personality disorder and dementia, and where the clinicians are treating primarily a psychiatric disorder rather than aggressive behaviour, (3) already prescribed an antipsychotic drug or any other drug that may interact with risperidone at the time of randomisation. A wash-out period of at least 2 weeks is required prior to randomisation, (4) any other contraindication for using risperidone including a previous history of severe adverse events, (5) has no fixed abode or any other reason for which compliance with trial medication and monitoring could pose a major problem, (6) is pregnant or trying to conceive, is breastfeeding, or is a woman of childbearing potential not using a highly effective birth control, (7) lactose intolerance, (8) known cardiovascular disease (e.g. heart failure, myocardial infarction, conduction abnormalities including family history of QT prolongation, dehydration, hypovolaemia, bradycardia), or electrolyte disturbances (hypokalaemia, hypomagnesaemia), or cerebrovascular disease, (9) a clinically significant low white blood cell count or a drug-induced leukopenia/neutropenia. and (10) a history of a confirmed neurogenic seizure in the last 3 months.

### Recruitment

We plan to recruit up to 50 adults with TBI who have been referred to a specialist service primarily for the management of aggressive behaviour. Participants will be recruited from outpatient clinics and hospital in-patient settings.

A patient who may be eligible for the study will be initially approached regarding the study by any healthcare professional who is involved in their care providing that the consultant for the team has agreed in principle to patients under their care taking part in the study.

If a healthcare professional has a patient under their care who they believe meets the eligibility criteria for this study, they will briefly introduce the study to them. Those patients who express an interest in taking part in the study and give verbal agreement to discuss their eligibility and possible enrolment into the trial with a member of the research team will be given a Study Information Sheet and a member of the research team will go through the Study Information Sheet with them, answering any questions. The patient’s agreement to be contacted by a researcher will be documented in part 1 of a Referral and Screening Form.

Potential participants will be given at least 24 h from receiving the Study Information Sheet to decide whether to take part in the trial. If the patient does decide to participate, they will be asked to complete a Consent Form and assigned a screening number. The date that the Study Information Sheet was given to the patient and the date of consent will be recorded. It will be made clear to the patient that they can withdraw at any time during the trial, without having to give a reason.

Written informed consent will include permission to notify the patient’s general practitioner (GP) about the enrolment of their patient into the trial, and to seek information about the patient from their named carer where possible. A copy of the signed Consent Form will be given to the patient. The original signed form will be retained in the Investigator Site File.

Study participants will be recruited from four centres in London and the South East of England: Imperial College NHS Healthcare Trust, South West London and St. George’s NHS Trust, Kent and Medway NHS Partnership Trust and St George’s University Hospitals NHS Foundation Trust.

### Screening

A pre-randomisation assessment of eligibility will be undertaken following consent by a check of the medical records for the patient that are accessible at the time of presentation to the specialist service. Where no reason for ineligibility has been identified, a discussion with the patient (and their carer where appropriate) will then take place to complete the eligibility check. Once eligibility is confirmed, the researcher will use the randomisation system to obtain a randomisation code for the participant. Following randomisation, the participant’s GP will be informed of their enrolment into the trial via letter.

### Sample size calculation

As this is a feasibility study we have not carried out any formal power calculation but estimate that it will be possible to recruit 50 patients over 18 months from the four centres. The sample size is also in keeping with recommendations on feasibility trials for other conditions [21–23].

### Study intervention, dosage and regime

A flexible oral dosing regimen of risperidone will be used. Dosing will start with 1 mg once daily and be titrated in 1-mg increments, not more than once every 7 days, to a maximum of 4 mg a day. The dose of risperidone in each capsule of active trial medication is 1 mg. The titration schedule will be as follows: (1) one capsule once daily (1 mg risperidone or matching placebo per day), (2) one capsule twice daily (2 mg risperidone or matching placebo per day), (3) two capsules at night and one capsule in the morning (3 mg risperidone or matching placebo per day), (4) two capsules twice daily (4 mg risperidone or matching placebo per day). The dose may also be reduced to a lower dose (minimum 1 mg once daily) at any time.

During the weekly telephone or in-person follow-up interview, the participant will complete the MOAS and be asked to answer questions on their health state and if they are experiencing any new or worsening side effects (documented and acted upon as an adverse event where reported). These data will be documented on a Trial Medication Review Form and this form passed to the participant’s prescriber (the principal investigator (PI) or other medically qualified individual to whom the task is delegated). The prescriber will decide whether to amend the dose based on the participant’s response to the trial medication as documented on this form.

We are using a lower dose of risperidone than is recommended in the *British National Formulary* (BNF) for the treatment of schizophrenia for the following reasons: (1) all the RCTs on intellectual disability and/or ASD used a relatively small dose of risperidone, (2) in practice clinicians use a low dose for treating aggression in TBI and (3) a recent animal study has shown that chronic use of high-dose risperidone as opposed to low-dose may affect cognitive function in TBI rodents [24]. Also there is a clinical belief (with no empirical evidence) that at low dose, risperidone may have an anti-arousal/anti-anxiety property which helps to improve aggression by ameliorating the underlying anxiety or arousal which may be triggering the aggressive behaviour.

### Concomitant medication

It would be unethical to restrict the therapeutic options of the clinical team; therefore, no restrictions will be imposed on the use of other treatments, except that those who remain in the trial will not be prescribed risperidone (aside from trial medication) and that prescribing of other medication should be done as if the patient were taking the active medication (for example, if they use an antipsychotic we will ask them to make sure that the overall daily dose of antipsychotics does not exceed the BNF-recommended daily chlorpromazine equivalent dose). As in clinical practice, medicines known to prolong the QT interval, medicines that modify CYP2D6 activity, or medicines strongly inhibiting or inducing CYP3A4 are prohibited.

Our approach will be primarily to record the use of all other medication, documenting details of dosage, and ensure the follow-up of all randomised participants, irrespective of the medication that they subsequently receive.

### Outcomes

#### Main outcome measure (baseline and weekly thereafter)

We will use the MOAS to assess improvement in aggression. The MOAS is a simple but widely used four-item scale that measures verbal aggression along with physical aggression towards other people, property and self. The MOAS is a valid and reliable instrument which has been used in many pharmacological intervention studies of aggression in different neuropsychiatric patient groups including TBI [19].

#### Other outcome measures (baseline and 12-week follow-up)

(1) Glasgow Outcome Scale-Extended version (GOS-E) [25], which is a widely used global outcome measure of social functioning following TBI, (2) Irritability Questionnaire (IRQ) [26], which has both a patient and a carer version and will assess the degree of underlying irritability behind aggressive behaviour. Some believe that in some people with TBI aggression is manifested as part of an episodic dyscontrol and these patients may respond better to a mood stabiliser. IRQ will help us to investigate any such differential treatment response, Clinical Global Impression-Improvement (CGI-I) and CGI-S (CGI-Severity) scales [27], (4) Udvalg for Kliniske Undersøgelser (UKU) scale [28] to assess the adverse effects of risperidone. If any abnormal movement is discovered then extrapyramidal adverse effects will be explored further and (5) two widely used quality of life (QoL) measures, namely the EuroQol five-dimension, five-level questionnaire ( EQ-5D-5L) [29] and the 12-item Short Form health survey (SF-12) [30], (6) mental health of the patient will be assessed using the Hospital Anxiety and Depression Scale (HADS) [31], which we have standardised for use in patients with TBI [32], (7) if available from the medical case notes at 12 weeks’ follow-up the following data will be collected; serum glucose/glycosylated haemoglobin (HbA1c), lipid profile, serum prolactin level, body weight and blood pressure.

We will use a proforma (Clinical Case Report Form, CCRF) specifically designed for this study to collect demographic data such as age, gender, etc. but also other relevant information such as timing, cause and severity of TBI, past and present history of physical and mental health problems and medication use, etc.

Where a carer has been identified and has consented to provide information, they will be asked about the participant’s level of aggression and irritability at 12-week follow-up, using the MOAS and IRQ, respectively (see Additional file 1 and Table 1).

**Table 1:** Assessment Schedule

Assessment	Baseline	1-week	2-week	3-week	4-week	5-week	6-week	7-week	8-week	9-week	10-week	11-week	12-week
MOAS	✓	✓	✓	✓	✓	✓	✓	✓	✓	✓	✓	✓	✓
Current health state questions	✓	✓	✓	✓	✓	✓	✓	✓	✓	✓	✓	✓	✓
MOAS (carer report) ^a^	✓												✓
IRQ	✓												✓
CGI	✓												✓
GOS-E	✓												✓
EQ-5D-5L	✓												✓
SF-12	✓												✓
UKU	✓												✓
HADS	✓												✓
CSRI	✓												✓
Concomitant medication	✓												✓
Basic clinical & psychiatric information^b^	✓												✓
Physical health measures	✓												✓
IRQ (carer report) ^a^	✓												✓
CWS ^a^	✓												✓

^a^where a carer is identified, and he/she consents to provide information

^b^where the data is already recorded in the participant’s medical records

### Carer contact

The participant will be asked to provide details of a non-professional carer if an outpatient, or identify a suitable member of nursing staff if an in-patient. Where such an individual is identified, they will be approached for consent to provide information on the participant’s level of aggression and irritability at baseline and 12-week follow-up, using the MOAS and IRQ, respectively. Non-professional carers will also be asked to complete the ‘Wellbeing’ section of the Carer Wellbeing and Support Questionnaire (CWS) in relation to their own health [33]. Patients who do not have a suitable carer may still take part in the study.

### Health economic data collection

We will use two health-related QoL measures, namely the EQ-5D-5L and the SF-12 in order to assess their suitability for data collection in this sample. If found useful in the feasibility study then one of these will be used in a future full-scale RCT for calculating Quality-adjusted Life Years (QALYs).

We will also measure service utilisation during the study period using the Client Service Receipt Inventory (CSRI) [34] at 12 weeks’ follow-up, which has been widely used in previous studies of health economics. This will allow us to capture non-medication-based interventions including neurorehabilitation received by the participants which may influence treatment outcome from risperidone. As some patients with TBI will suffer from memory problems, they may not be able to complete all the sections in the CSRI, and information may need to be gathered from their carers or from clinical records. If that is the case, we will need to assess which items can be collected from records, which will require input from informants and whether there will be enough informants available for the full RCT for this purpose. It will not be possible to carry out a full cost-effectiveness analysis in this feasibility study. However, we will calculate indicative costs using descriptive statistics.

### Follow-up

We will assess participants at 12 weeks. We expect the dose of risperidone to be stabilised within the first 3 weeks and its effect established within another 3 weeks so that outcomes could be assessed at 12 weeks. We will address the reasons for dropout to ensure that they are minimised for the full RCT.

### Randomisation procedures

Remote web-based randomisation will be undertaken through a fully automated service operated by an external organisation ‘Sealed Envelope’. We will use random permuted blocks stratified by study centre. Equal numbers of participants will be randomised to risperidone and placebo. Randomisation of a participant at a site will generate a randomisation code that corresponds to trial medication held in the local pharmacy at the site where the participant was recruited.

### Concealment and unblinding procedure

Trial medication will be identified by a randomisation code that corresponds to either risperidone or placebo, labelled during manufacture and bottling of the capsules. This will keep the patients, clinicians, carers and researchers blind to the allocation of active drug and placebo. Three bottles of trial medication will have the same randomisation code to allow sufficient supply for one participant to complete 84 consecutive days. Trial medication will be supplied to site pharmacies in blocks sufficient to provide medication to six participants. There will be a 24-h telephone number available for the clinicians and research team if the randomisation code needs to be broken. This information will help to design pharmacy arrangement and associated logistics for the full RCT which is likely to involve a large number of centres.

### Treatment procedures

Once randomisation has taken place, a study prescription form containing the patient’s details (including their randomisation code) will be signed by the site PI or the patient’s usual prescriber (another medically qualified individual to whom the task is delegated), and sent to the study site pharmacy. This prescription will allow for the local site pharmacy to give the first and subsequent bottles of trial medication to the participant (no further prescription will be necessary for the second and third bottles of trial medication from the medication pack to be given out later in participation). An appropriate bottle of trial medication will be selected and prepared for collection.

Where the participant is an in-patient, the trial medication will be delivered to their ward, with the prior consent of the ward manager. For in-patients, arrangements will be made for the trial medication to be given by ward staff in the same way as other prescribed medication would be in this setting and it written up on the participant’s drug chart.

Patients will be left with a telephone number for the local research contact for any queries or to report any suspected adverse effects. Someone from the research team will telephone on the two consecutive days after starting the treatment and weekly thereafter. Participants will be asked to return all unused medication, which will allow us to check for compliance.

### Statistical analyses

As this is a feasibility study, any hypothesis testing at this stage will be considered preliminary as the study is not adequately powered. Instead we will work out the standard deviation of the MOAS score at 12 weeks’ follow-up and as changes from baseline. These data will inform a sample size calculation for our proposed full-scale RCT in future, along with any other relevant published information.

As this is a feasibility study we will only calculate 95% confidence intervals of score changes in outcome measures from baseline to follow-up. We will ensure that the way data are collected allows us to estimate Number Needed to Treat (NNT) in the full RCT, which will use an intention-to-treat analysis.

We will work out the range and mean score change in MOAS from baseline to 12 weeks for those who have scored ‘improved’ and ‘very much improved’ according to the CGI-I scale. This information will determine what effect size on MOAS corresponds to a clinically important improvement for an individual patient. This will inform the sample size calculation for the full RCT.

### Post-trial Investigational Medicinal Product (IMP) arrangements

Following a participant’s 12-week assessment, an email will be sent to the referring clinician directly from the randomisation system, informing them of the participant’s trial arm allocation. Where a participant has completed the participation period in full, this will allow for arrangements to be made for the participant to continue on risperidone if appropriate and desired. No further risperidone will be supplied by the sponsor. The participant will be advised to contact their clinician to discuss their trial arm allocation if they wish to know whether they were taking the active or the placebo medication.

### Assessment of participant compliance

Compliance includes both adherences to IMP and protocol procedures. Non-compliance to the protocol or trial procedures should be documented by the investigator in the participant CRF. Non-compliance data will be collected as part of the assessments. This will include noting missing assessment data and dating assessments to determine when these occurred outside the study visit window. In addition, the medication diary cards will be collected from the participants and used as a measure of medication adherence. However, as this is a feasibility study, no threshold criteria have been set for patient withdrawal due to non-compliance.

### Recording and reporting adverse events and reactions

Notification and reporting adverse events/reactions in this clinical trial will be done according to procedures described in Imperial College Healthcare Good Clinical Practice.

### Process evaluation

A parallel process evaluation using both qualitative and quantitative methods will examine trial recruitment and the feasibility and acceptability of trial procedures from clinician and patient perspectives. The quantitative component of the evaluation will involve sending out bespoke questionnaires designed for the study to participants, their carers and the clinicians to collect data on treatment adherence, non-pharmacological interventions including psychological therapies and other rehabilitation programmes, obstacles for recruitment and retention, and support needed for the clinicians, patients and carers.

Semi-structured qualitative interviews will be conducted with clinicians in each of the four sites. These will be supplemented by semi-structured interviews with a sample of 10 trial participants and/or their carers purposively sampled to represent site and treatment allocation.

The interview guides used in each context will be developed as the feasibility study progresses and will be sensitive to emergent themes. These interviews will be audio-recorded, transcribed and analysed using a thematic framework approach and managed using NVivo [35] computer software. An initial framework will be based on the study aims of how feasible it is to undertake a definitive trial. This will be further developed using analytic induction from early interviews and iteratively revised as data collection and analysis progress.

### Project management

A Combined Independent Oversight Committee will be in place prior to the start of the study and include an independent Chair and at least two other independent members. This committee will provide overall supervision of the trial and ensure that it is being conducted in accordance with protocol and current legislation. It will also review trial data in order to identify patterns in the data that may suggest the need to halt the trial.

A Trial Management Group will also be set up prior to the start of the study, and will include those individuals responsible for the day-to-day management of the trial, such as the chief investigator (CI), representative PI(s) and trial management staff. In addition, a research assistant and individual(s) who is/are able to contribute a patient and/or wider public perspective will be included. The role of the group will be to monitor all aspects of the conduct and progress of the trial, ensure that the protocol is adhered to and take appropriate action to safeguard participants and the quality of the trial itself. The group should consider and act on the recommendations of the Combined Independent Oversight Committee, the Medicinal Health Regulatory Authority (MHRA) and the Research Ethics Committee (REC).

We will set up a Patient Advisory Group which will meet regularly and report back to the Trial Management Group through one co-applicant who will also sit on the Trial Management Group. The Patient Advisory Group will also advise on accessible information leaflets, Consent Forms and web-based newsletters for the project. Additionally a patient co-applicant will be part of the Trial Management Group.

### Access to source data/documents

The investigator(s)/institution(s) will permit trial-related monitoring, audits, REC review, and regulatory inspection(s), providing direct access to source data/documents. Trial participants are informed of this during the informed consent discussion. Participants will consent to provide access to their medical records.

### Ethics and regulatory requirements

The sponsor will ensure that the trial protocol, Study Information Sheets, Consent Forms, GP letter and submitted supporting documents have been approved by the MHRA and a main REC prior to any patient recruitment. The protocol and all agreed substantial protocol amendments will be documented and submitted for ethical and, where necessary, regulatory approval prior to implementation according to sponsor-specific Standard Operating Procedures (SOPs).

Before the site can enrol patients into the trial, the Trust Research and Development (R&D) for the site must grant written permission. It is the responsibility of the PI at each site to ensure that all subsequent amendments gain the necessary approval. This does not affect the individual clinician’s responsibility to take immediate action if thought necessary to protect the health and interest of individual patients. All PIs will be required to have an up-to-date Good Clinical Practice training certificate.

Within 90 days after the end of the trial, the CI/sponsor will ensure that the main REC and the MHRA are notified that the trial has finished. If the trial is terminated prematurely, those reports will be made within 15 days after the end of the trial. The CI will supply the sponsor with a summary report of the clinical trial, which will then be submitted to the MHRA and main REC within 1 year after the end of the trial.

### Data handling and analysis

It will be the responsibility of the investigator as delegated to ensure the accuracy of all data entered in the CRFs. The delegation log will identify all those personnel with responsibilities for data collection and handling, including those who have access to the trial database.

Data will be collected using paper CRFs and then transcribed onto a Microsoft Access database that is created prior to the start of the trial. Data monitoring will be carried out according to the trial-specific monitoring plan. This details the quality control checks to be carried out during site visits and when checking the database. All serious adverse event and primary outcome data will be checked and a random sample of the secondary outcome data. The database will be stored on a network drive at Imperial College London, which is backed up daily. Data will only be input onto the database at Imperial College London from a computer owned by the organisation that has access to the network drive.

At the end of the trial, data monitoring will be completed and the database ‘locked’ so that no further data entry is possible. At this point the data will be given to the trial statistician along with the randomisation list stating whether participants were allocated to arm A or arm B. The data will be analysed without knowledge of which arm relates to the active trial medication.

### Monitoring requirements for the trial

A trial-specific monitoring plan will be established based on the trial risk assessment. The trial will be monitored according to the agreed plan.

### Dissemination

Dissemination will be ongoing. A project website at Imperial College London hyperlinked with the Royal College of Psychiatrists, research network and relevant service users’ organisations, will host relevant information. A newsletter will be distributed twice a year to update interested parties on the progress of the project and the findings of each stage. It will also maintain interest in the project and help facilitate recruitment. Careful attention will be paid to providing accessible information for TBI patients. Accessible newsletters will be distributed via service-user organisations and the final guides and reports will be available in accessible format.

One conference will be organised in London at the Royal College of Psychiatrists’ conference venue at the end of the project to disseminate findings. This event will be free of charge to attend and open to a wide range of delegates. A summary report will be produced and sent to a wide range of stakeholders. Papers will be prepared for publication in high-impact peer-reviewed journals. Findings of the study will also be presented in local, national and international meetings and conferences. The results of the trial will be posted by the sponsor on EudraCT and made available to the public via the EU Clinical Trials Register. Care will be paid to disseminating to care staff and family carers via appropriate organisations such as Headway. If requested, each study participant will be sent a personal letter thanking them for their involvement in the study which will also provide a short summary of the study findings and details of how further information about the project can be obtained.

## Discussion

Aggressive behaviour causes immense distress among people with TBI and their families, sometimes leading to family breakdown. This is a hidden disability within society as many of these patients suffer from lack of appropriate intervention and service provision. These patients believe that an effective intervention is urgently needed to help improve their quality of life. An appropriately designed RCT will provide much needed evidence to support a particular type of intervention such as drug therapy.

Each year 1.4 million people attend Accident and Emergency departments in England and Wales with TBI and a high proportion of them develop long-lasting consequences including irritable and aggressive behaviour [1–6]. This is proving to be a major burden for the NHS. A clinically and cost-effective intervention will reduce this burden on the NHS for which appropriately conducted RCTs are mandatory. This also has the potential to reduce the need for mental health service, in-patients and emergency departments, where these patients are likely to present in the absence of an appropriate intervention.

Despite the high prevalence of neuropsychiatric and neurobehavioural problems after TBI [7–9], these patients often do not receive appropriate treatment and in most cases do not receive any treatment largely because of lack of awareness of these problems within the medical fraternity but also because of lack of appropriate services [36]. As a result the neuropsychiatric sequelae of TBI result in ‘hidden disability’ in which patients and their families suffer in silence in the absence of any meaningful intervention.

One reason for carrying out this feasibility study is to assess the rate of recruitment and attrition as this sort of RCT has never been carried out involving this patient group in the UK or elsewhere in the world for that matter [10–12]. Therefore, there may be an expected degree of apprehension on the part of patients, their carers and the practicing clinicians to take part in a blinded study which will include placebo as a possible intervention [37]. However, our initial discussion with patient groups and clinicians indicates that many of them are willing to take part in such a study. As this is completely uncharted territory (as no large-scale properly designed RCT exists) [38], it is necessary to carry out a feasibility study like the one we are proposing before embarking on a full-scale RCT.

This feasibility study will assess the willingness of the clinicians to recruit into the study in practice, among a group of clinicians who have already agreed in principle to recruit. Issues around recruitment will be explored, such as any obstacle that can be addressed, and any procedure that can be used to identify potential participants. The feasibility study will also help to refine our estimates of the number of eligible patients for recruitment in each centre, which will allow us subsequently to determine how many centres we need to recruit from for the full RCT.

Apart from the information mentioned earlier on recruitment rate, retention, etc., this feasibility study will also inform whether randomisation is possible for a future definitive RCT, what factors should be considered for clustering, how to stratify the sample, applicability of the outcome measures, deciding on primary and secondary outcome measures, acceptability of the intervention and if any revision is needed for their future application. and to determine what should be an ideal follow-up period. During the study we shall also network widely with organisations providing service for patients with TBI in order to assess whether recruitment for a required number of participants is possible in a future definitive RCT [22]. We shall also network with other professionals working in the field in order to involve them in a future RCT if necessary.

Success of this project will be evident in completion of the study as per timetable. The principal challenges will be recruitment of the study sample, maintaining engagement with treatment and minimising loss to follow-up. Should the rate of recruitment or follow-up be below that stated in the proposal we will consider available strategies [23]. We will also explore the possibility of recruiting from other centres within the consortium as a large-scale definitive RCT is urgently needed in this field in order to develop more effective responses to the challenging problem of management of aggression in patients with TBI.

## Additional file

Additional file 1: SPIRIT Checklist. (DOC 121 kb)

## Abbreviations

AE: Adverse event
AR: Adverse reaction
CI: Chief investigator
CRF: Case Report Form
CSRI: Client Service Receipt Inventory
CWS: Carer Wellbeing and Support Questionnaire
DMC: Data Monitoring Committee
DSUR: Development Safety Update Report
GCP: Good Clinical Practice
GOS-E: Glasgow Outcome Scale-Extended
ICF: Informed Consent Form
IMP: Investigational Medicinal Product
ISRCTN: International Standard Randomised
MOAS: Modified Overt Aggression Scale
PI: Principal investigator
RCT: Randomised controlled trial
SAE: Serious adverse event
SOP: Standard Operating Procedure
TMG: Trial Management Group
